# Impact of Seton Use on Clinical, Patient-Reported, and Healthcare Resource Utilization Outcomes in Complex Crohn’s Perianal Fistulas: A Systematic Literature Review

**DOI:** 10.1093/ibd/izae186

**Published:** 2024-09-19

**Authors:** Ian White, Chitra Karki, Parnia Geransar, Lilia Leisle, Sophia Junker, Phillip Fleshner

**Affiliations:** Department of Surgery, Beilinson Hospital, Rabin Medical Center, Petah Tikva, Israel; Faculty of Medicine, Tel Aviv University, Tel Aviv, Israel; Takeda Pharmaceuticals USA, Inc., Cambridge, MA, USA; Takeda Pharmaceuticals International AG, Zurich, Switzerland; Ingress-Health HWM GmbH, an affiliate of Cytel, Inc., Real World & Advanced Analytics, Berlin, Germany; Ingress-Health HWM GmbH, an affiliate of Cytel, Inc., Real World & Advanced Analytics, Berlin, Germany; Cedars-Sinai Medical Center, Division of Colon and Rectal Surgery, Los Angeles, CA, USA

**Keywords:** Crohn’s disease, perianal fistula, seton

## Abstract

**Background:**

Optimal treatment strategies for seton use in patients with Crohn’s perianal fistulas (CPF) remain elusive. This systematic literature review aimed to summarize clinical, patient-reported, and healthcare resource utilization (HCRU) outcomes associated with seton use for symptomatic relief and treatment of complex CPF.

**Methods:**

Electronic databases (MEDLINE, Embase, EBM Reviews, EconLit) were searched. Titles, abstracts, and relevant full texts were screened by 2 reviewers for inclusion using prespecified PICOS-T criteria. Articles published in English between January 1, 1980 and September 6, 2021 were included; animal/in vitro studies and case reports with <5 patients were excluded. Outcomes of interest included rates of complete response/remission and fistula recurrence in patients receiving seton with/without infliximab or biologics. Data were summarized using descriptive statistics.

**Results:**

Overall, 56 studies were included (full texts: *n* = 43; congress abstracts: *n* = 13). CPF and clinical outcome definitions were heterogeneous. Rates (range) of complete response/remission varied widely (seton: 13%-75%; seton + infliximab: 23%-100%; seton + biologics: 23%-59%) as did rates for fistula recurrence (seton: 4%-68%; seton + infliximab: 0%-50%; seton + biologics: 0%-17%). Rates of fistula-related reintervention, new fistula or abscess formation, and abscess recurrence were also varied; more consistency was observed regarding the use of patient-reported outcomes. Few studies reported outcomes from pediatric/adolescent patients or HCRU.

**Conclusions:**

Optimal use of seton in patients with CPF remains unclear. International standardization of definitions for CPF and related clinical outcomes are required to permit data comparability and identify the most effective treatment strategies involving seton use in CPF.

## Graphical Abstract

**Graphical Abstract:**
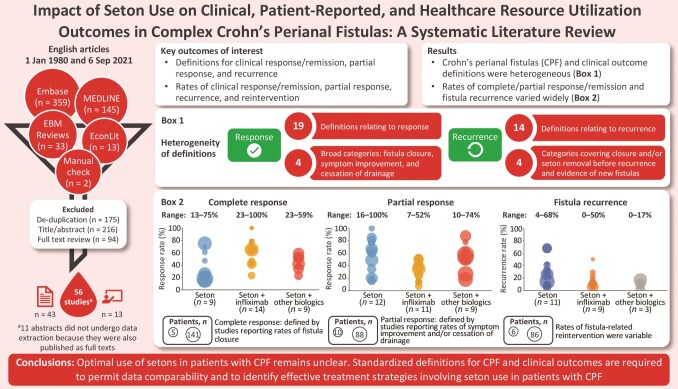

**Key Messages**

- What is already known?

Setons are often used as a bridge to surgical interventions and can also be used for long-term fistula management in patients with complex Crohn’s perianal fistulas (CPF).

- What is new here?

This study is the first to systematically review and evaluate the effect of seton use on clinical, patient-reported, and healthcare resource utilization outcomes in patients with CPF and identified heterogeneous reporting regarding outcomes associated with seton use in this patient population.

- How can this study help patient care?

This study highlights that, in order to develop optimal seton-based treatment strategies, there is a need for internationally recognized, consistent definitions for CPF and clinical outcomes, as well as a need to develop standardized CPF study designs.

## Introduction

Crohn’s disease (CD) is a type of inflammatory bowel disease that primarily affects the gastrointestinal tract.^1^ Crohn’s perianal fistulas (CPF) are a devastating complication of CD, and can cause pain and drainage of pus, blood, gas, or stool from the fistula openings.^2–4^ The cumulative incidence of CPF in patients with CD is estimated to be 11%-15%, 16%-21%, and 26%-28% at 5, 10, and 20 years, respectively^5–8^; they can significantly impair a patient’s health-related quality of life (QoL), including their psychological well-being, and place a considerable, and likely underestimated, economic burden on healthcare systems.^3,9–12^ Currently, a consensus for classifying CPF is lacking; however, in clinical practice, most experts use a classification of “simple” or “complex.”^13,14^ Simple CPF are superficial, low intersphincteric, or low trans-sphincteric fistulas.^13^ Complex CPF are characterized by high intersphincteric/trans-sphincteric tracts or extrasphincteric/suprasphincteric tracts and may have multiple external openings.^13^ More recently, CPF classification has focused on clinical symptoms rather than anatomical position to aid in the standardization of research and clinical practice.^15^ The treatment of complex CPF is challenging, requiring a multidisciplinary approach for optimal patient management and the use of both medical and surgical interventions.^16–19^ Ultimately, the treatment goals are sustained fistula closure, preservation of fecal continence, and alleviation of symptoms.^2,20^ Regardless of the treatment strategy employed, loose or non-cutting setons are often used in the treatment of complex CPF to help control perianal sepsis and prevent recurrent abscess formation.^21^ Although setons are often used as a bridge to other treatment interventions, they are also used as a long-term intervention for complex CPF, maintaining fistula patency, facilitating healing, and potentially improving patient QoL.^21^ Premature seton removal can increase the risk of abscess formation, fistula recurrence, delayed healing, and other potential complications^22,23^; however, further evidence is needed to develop guidance on the optimal timing of seton removal.^24^ This systematic literature review (SLR) aimed to evaluate the available evidence on clinical, patient-reported, and healthcare resource utilization (HCRU) outcomes associated with seton use for the management complex CPF.

## Methods

This SLR was conducted according to the guidelines of the Cochrane Collaboration (London, UK), the Centre for Reviews and Dissemination (CRD) (York, UK), and the National Institute for Health and Care Excellence (NICE) (London, UK) for evidence synthesis using a prespecified study protocol.^25–27^

### Data Sources, Search Strategy, and Study Selection

A systematic search of electronic databases (MEDLINE, Embase, EBM Reviews, and EconLit) was conducted for relevant articles published between January 1, 1980 and September 6, 2021 using predefined search algorithms. To avoid missing relevant information owing to varied terminology for complex CPF, the literature search was conducted for “anal, perianal, and rectal fistulas,” without the specification term “complex.” A manual bibliography check of included articles was also conducted at the full-text screening stage, and all identified review articles, guidelines, SLRs, or meta-analyses were screened for relevant original publications not identified by the electronic database search. No manual search of conference proceedings was required as electronic database searches covered key conference proceedings, which include:

- American College of Gastroenterology (ACG)
- American Society of Colon and Rectal Surgeons (ASCRS)
- Advances in Inflammatory Bowel Diseases (AIBD)
- Crohn’s & Colitis Congress (CCC)
- Digestive Disease Week (DDW)
- European Crohn’s and Colitis Organisation (ECCO)
- European Society of Coloproctology (ESCP)
- United European Gastroenterology Week (UEGW).

Titles and abstracts were assessed independently by 2 reviewers for inclusion using the population, interventions, comparators, outcomes, study designs, and time (PICOS-T) selection criteria (Supplementary Table 1). Full-text versions of the included articles were screened independently by 2 reviewers to assess study eligibility; any disagreement between reviewer decisions was discussed and, if necessary, a mediator was involved in taking a final decision.

### Inclusion and Exclusion Criteria

Included articles were those published in English, describing any clinical trial, observational study, or case report with at least 5 patients in whom seton placement was used for symptomatic relief and treatment of complex CPF. Outcomes of interest included study and patient characteristics, definitions for and rates of clinical response/remission, partial response, and recurrence, alongside rates of reintervention. Impacts of seton use on patient-reported outcomes (PROs) and HCRU were also assessed. Further details of outcomes of interest are included in Supplementary Table 1. SLRs, meta-analyses, review articles, and guidelines were included for bibliography checks only. Animal/in vitro studies and case reports with fewer than 5 patients were excluded.

### Data Extraction

Two independent reviewers followed an agreed data extraction sheet. Data were extracted for patients with complex CPF (where specified), for mixed populations of complex and simple CPF, and for patients without a clear description of the fistula’s nature. For the purposes of data extraction, complex CPF were defined as having met at least one of the following criteria:

- high intersphincteric, trans-sphincteric, extrasphincteric, or suprasphincteric origin of the fistula tract
- presence of at least 2 external openings (tracts)
- associated collections.

### Quality Assessment

The scientific quality of the included studies was assessed using different criteria depending on the study type; randomized controlled trials (RCTs) were assessed using criteria recommended by NICE,^25^ whereas for non-RCTs and observational studies, the criteria recommended by the Risk Of Bias In Non-randomized Studies – of Interventions (ROBINS-I) tool were used.^28^ Conference abstracts did not undergo quality assessment owing to their limited amount of data availability. Quality assessments were conducted independently by 2 reviewers. Any conflicting decisions were resolved after discussion between the 2 reviewers or via the inclusion of a third reviewer.

### Data Analysis

For the purposes of data analysis, response definitions were grouped into 3 categories: fistula closure, improvement of symptoms, and cessation of drainage. Complete response/remission was defined by the studies reporting rates of fistula closure. Rates of symptom improvement were considered as a partial response, as was cessation of drainage in most cases. Where rates of symptom improvement and cessation of drainage were reported in a single study, rates of cessation of drainage were considered as a complete response/remission. Where studies did not define a response rate, responses were considered as partial. Categorical variables were extracted as *n* (%) and continuous variables were extracted as mean, SD, SE, median, and min/max values. Where possible, data were presented by intervention: seton treatment only, seton treatment with infliximab (IFX), and seton treatment with biologics (including any anti-tumor necrosis factor [TNF]-α treatment other than IFX alone). Data presented for combinations of seton with biologics excluded outcomes from treatment with seton and IFX only. All data were reported using descriptive statistics.

## Results

### Identified Studies

#### Publication, study, and patient characteristics

**Figure 1.**
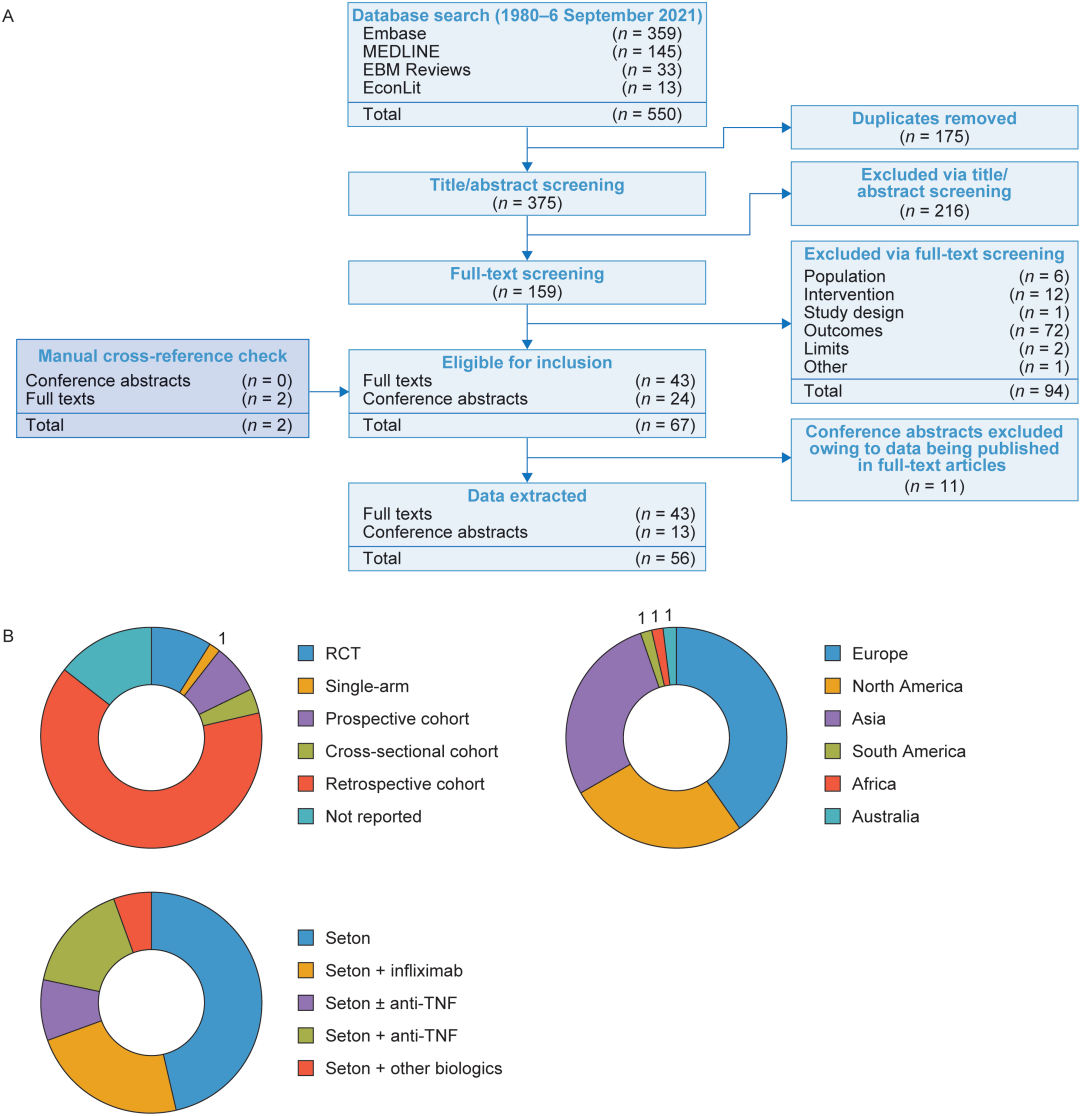
A, Preferred Reporting Items for Systematic Reviews and Meta-Analyses (PRISMA) flow chart. B, Study characteristics. Anti-TNFs include adalimumab and certolizumab. Other biologics include ustekinumab and vedolizumab. Numbers inside each chart indicate study count. Abbreviations: RCT, randomized controlled trial; TNF, tumor necrosis factor.

In total, 56 studies^29–84^ were included, comprising 50 observational studies and 6 registered clinical trials (Figure 1A). Of these, 25 assessed outcomes in patients with both complex and simple CPF, 16 in patients with complex CPF only, and 15 did not report the fistula type. Some studies included specific definitions for complex CPF (*n = *14); the most commonly used classifications were the Parks^40–42,48,52^ and American Gastroenterological Association (AGA) classifications^35,52,64,69,73^ (*n = *5 each), whereas 4 studies used their own definitions.^53,56,66,77^ Median follow-up times varied between studies, ranging from 7.5 months^62^ to 79 months^80^ and 3 months^74^ to 18 months^81^ for observational studies and RCTs, respectively. Similarly, participant numbers were also highly variable, with as few as 5 participants being recruited to one study^73^ and as many as 326 participating in another study.^65^ Additionally, substantial variability was observed in patient baseline characteristics and disease states (Table 1). The treatments for CPF evaluated in the studies included seton placement alone and seton placement in combination with an anti-TNF (IFX, adalimumab, certolizumab) or other biologics (ustekinumab, vedolizumab) (Figure 1B); however, few studies directly compared the clinical outcomes of seton placement only and seton placement plus an anti-TNF (*n = *5^37,41,46,66,81^). Full details of the characteristics of the studies included in this SLR are presented in Supplementary Tables 2 and 3.

**Table 1.** Baseline characteristics and disease state.

Baseline characteristic/disease state	Number of studies	Range (median)
Mean age, y	56	13.3-43.0
Male	56	27%-92% (64%)
Duration of seton use, wk	23	4-380
CD duration at index, y	10	0-11
Smoker	10	6%-44% (22%)
Baseline CDAI score (mean or median)	9	74-180
Baseline PDAI score (mean or median)	9	3-11
Duration between CD diagnoses and CPF, mo (mean)	3	6.7-54
Overall disease state		
Newly diagnosed	2	N/A
Recurrent	2	N/A
Mixed (new or recurrent)	14	N/A
Not reported	38	N/A

Abbreviations: CD, Crohn’s disease; CDAI, Crohn’s Disease Activity Index; CPF, Crohn’s perianal fistulas; N/A, not applicable; PDAI, Perianal Disease Activity Index.

### Quality Assessment

The scientific quality of all included full-text articles (*n = *43) was reviewed. Overall, 39 observational studies and 1 nonrandomized clinical trial were assessed using the ROBINS-I tool, of which most (*n = *20) had a moderate risk of bias, mostly due to confounding and bias in classification of intervention. The remaining had either a low (*n = *4), serious (*n = *15), or critical (*n = *1) risk of bias. Serious risk of bias was due to confounding and bias in the measurement of the outcomes. Of the 6 RCTs, 3 were assessed for risk of bias using the NICE methodology checklist for RCTs. Of these, 2 were considered to have a moderate risk of bias and 1 had a high risk of bias. Owing to a lack of sufficient information to complete assessments fairly, conference abstracts (*n = *13) were not evaluated.

### Duration of Seton Placement and Optimizing the Timing of Seton Removal

Of the included studies, fewer than half (*n* = 23, 41%^30,35,38,42,44,47,48,51,53,54,56,58,60,61,67–69,73,75,76,81–83^) quantified the duration of seton placement in patient populations. For patients receiving seton placement only (*n* = 16 studies), durations ranged from a median of 6 months^60,68,82^ to 31.5 months,^53^ with some studies reporting durations as short as 1.4 months^56^ and as long as 88 months.^82^ For those receiving seton plus any biologic, the duration of seton placement was similarly variable, with average values ranging from 6 months^51^ to 31.2 months,^61^ and studies reporting durations ranging from 1 month^51^ to 68.4 months.^61^ In total, 8 studies reported the timing of seton removal during anti-TNF treatment, which in most cases ranged from 6 to 12 months after placement; however, one study reported seton removal after only 1.4 months,^81^ whereas another reported seton placement of 31.2 months.^61^ In addition, 4 studies^59,77–79^ reported the timing of seton removal in relation to anti-TNF treatment, with removal typically occurring between the second and fourth anti-TNF infusion.^59,77,79^ This is aligned with the general experience, noted in one study, that fistulas begin to close around the seton 0.5-0.9 months after the second anti-TNF infusion^59^; however, one study did report seton removal in some patients beyond the fourth anti-TNF infusion.^78^

Overall, there was a paucity of data with respect to the criteria used to determine the optimal time for seton removal. One study noted that seton drainage of less than 7.9 months was significantly associated with sustained fistula closure in patients treated with seton plus IFX.^35^ One study highlighted that patients undergoing imaging assessment (magnetic resonance imaging [MRI] or transrectal ultrasound) prior to seton removal had a significantly lower recurrence rate compared with those not undergoing imaging techniques (27% vs 68%, *P *=* *.005), suggesting that imaging may help to determine the optimal timing of seton removal.^39^ Finally, for pediatric patients, use of the Pediatric Crohn’s Disease Activity Index (threshold score < 10) was noted as a potential tool for guiding seton removal.^40^

### Clinical Outcomes

#### Complete response/remission and partial response

**Figure 2.**
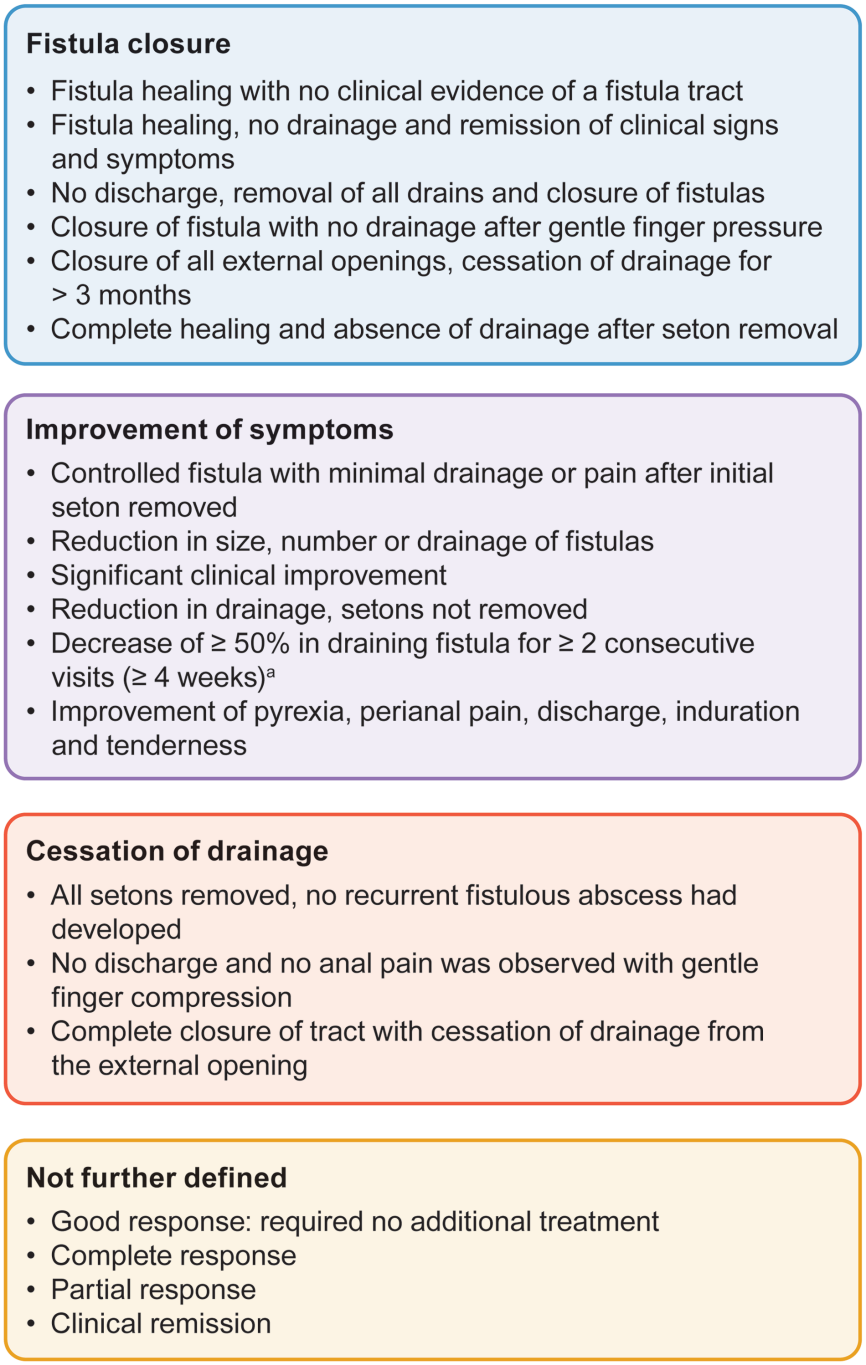
Reported definitions of response/remission in patients with complex Crohn’s perianal fistulas. ^a^According to the Fistula Drainage Assessment Index.

**Figure 3.**
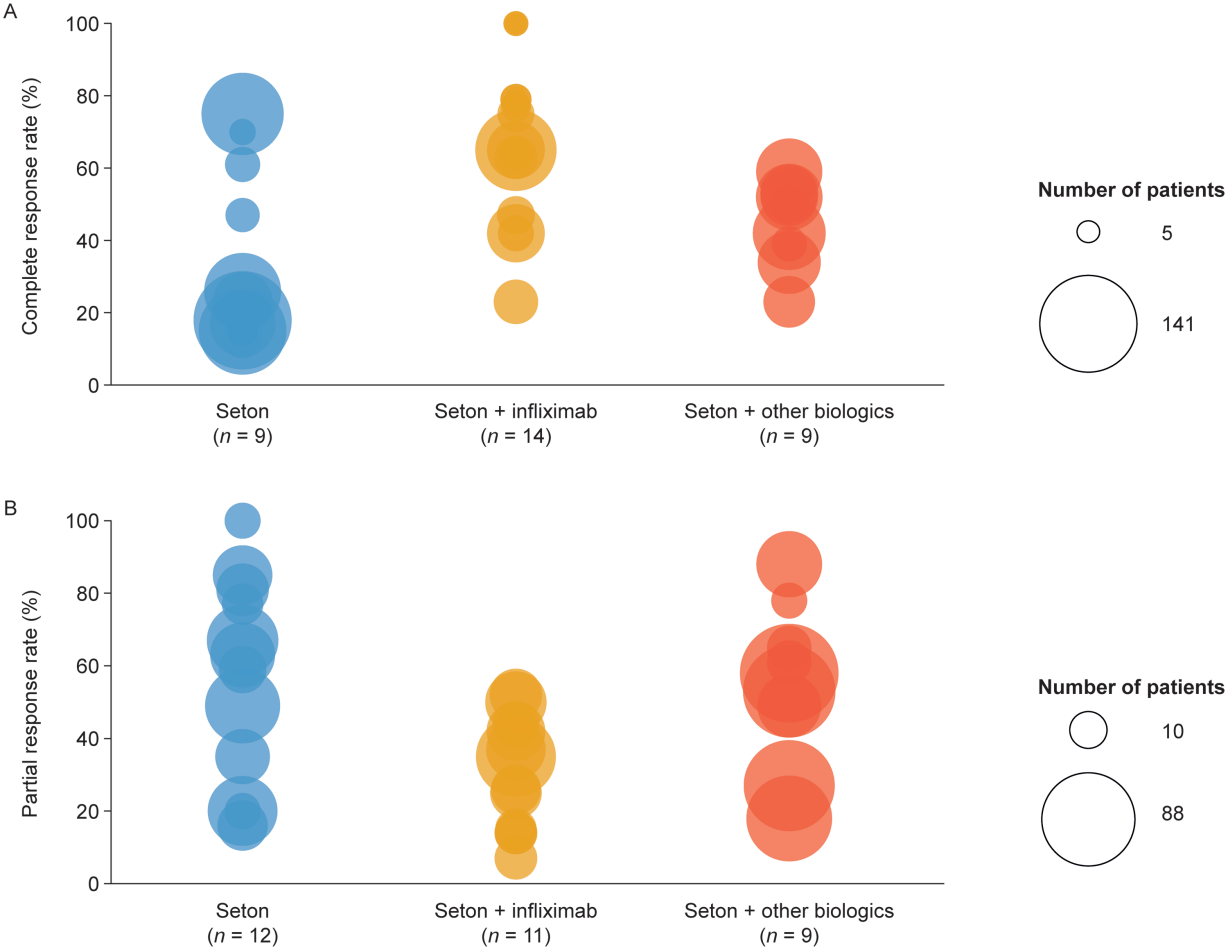
Complete (A) and partial (B) response rates to treatment with seton placement only, seton plus infliximab, and seton plus biologics. Response rates from complex and mixed (or non-reported) Crohn’s perianal fistula patient populations have been combined and plotted as circles for each intervention. Each circle represents a complete or partial response rate from a single study, with the size of each circle representing the number of patients investigated in the respective study. The minimum and maximum number of patients are shown in the legend.

In total, 54 studies reported on clinical outcomes, of which 41 reported complete response/remission or partial response rates (Supplementary Table 4). The primary mechanism for determining response was via physical examination; however, 9 studies described radiological techniques that were used to assess response.^33,39,44,51,64,66,70,74,75^ Definitions for complete response/remission or partial response were heterogeneous across studies and were grouped into 3 categories (fistula closure, cessation of drainage, and improvement of symptoms) to reduce complexity (Figure 2). Rates of complete response/remission were varied both within and between interventions, ranging from 13%^83^ to 75%^62^ for patients receiving seton only (*n = *9 studies), 23%^69^ to 100%^30,59^ for patients receiving seton placement and IFX (*n = *14 studies), and 23%^56^ to 59%^29^ for those receiving seton and biologics (*n = *9) (Figure 3A). The majority of studies (*n = *18/29; 62%) reported that all patients had their seton removed prior to achieving complete response. One study reported that a third of patients (5/15) did not want setons to be removed even after achievement of complete response (defined as cessation of drainage and an absence of anal pain, despite retention of the seton) due to anxiety regarding abscess recurrence.^47^ The remaining 10 studies provided no information regarding seton removal (Supplementary Table 4). Consistent with the results observed for complete response, rates of partial response also were varied, ranging from 16%^54^ to 100%^82^ for patients treated with seton only (*n = *12 studies), 7%^43^ to 52%^77^ for patients receiving seton placement and IFX (*n = *11 studies), and 10%^51^ to 74%^72^ for patients receiving seton with biologics (*n = *9 studies) (Figure 3B). Only 3 studies compared response rates between seton placement only, seton placement combined with IFX, or seton placement combined with biologics^37,41,66^; of these, 2 studies reported significantly higher clinical response rates in patients treated with seton placement and IFX when compared with those receiving seton placement alone (~17% for seton vs ~42% for seton plus IFX^41^ and ~20% for seton vs ~65% for seton plus IFX/adalimumab,^37^ respectively). The remaining study reported that those receiving seton with IFX had a significantly longer time to relapse than those treated with seton only (3.6 ± 0.5 months for seton vs 10.1 ± 2.4 months for seton plus IFX).^66^ There was a paucity of data with respect to the time to response, with only 5 studies reporting this outcome^52,53,57,73,77^ and even fewer (*n = *1)^83^ reported of duration of response. Time to any response after treatment with seton plus IFX ranged from 14 weeks (median)^57^ to 37 months (mean).^57,73^

#### Fistula recurrence

**Figure 4.**
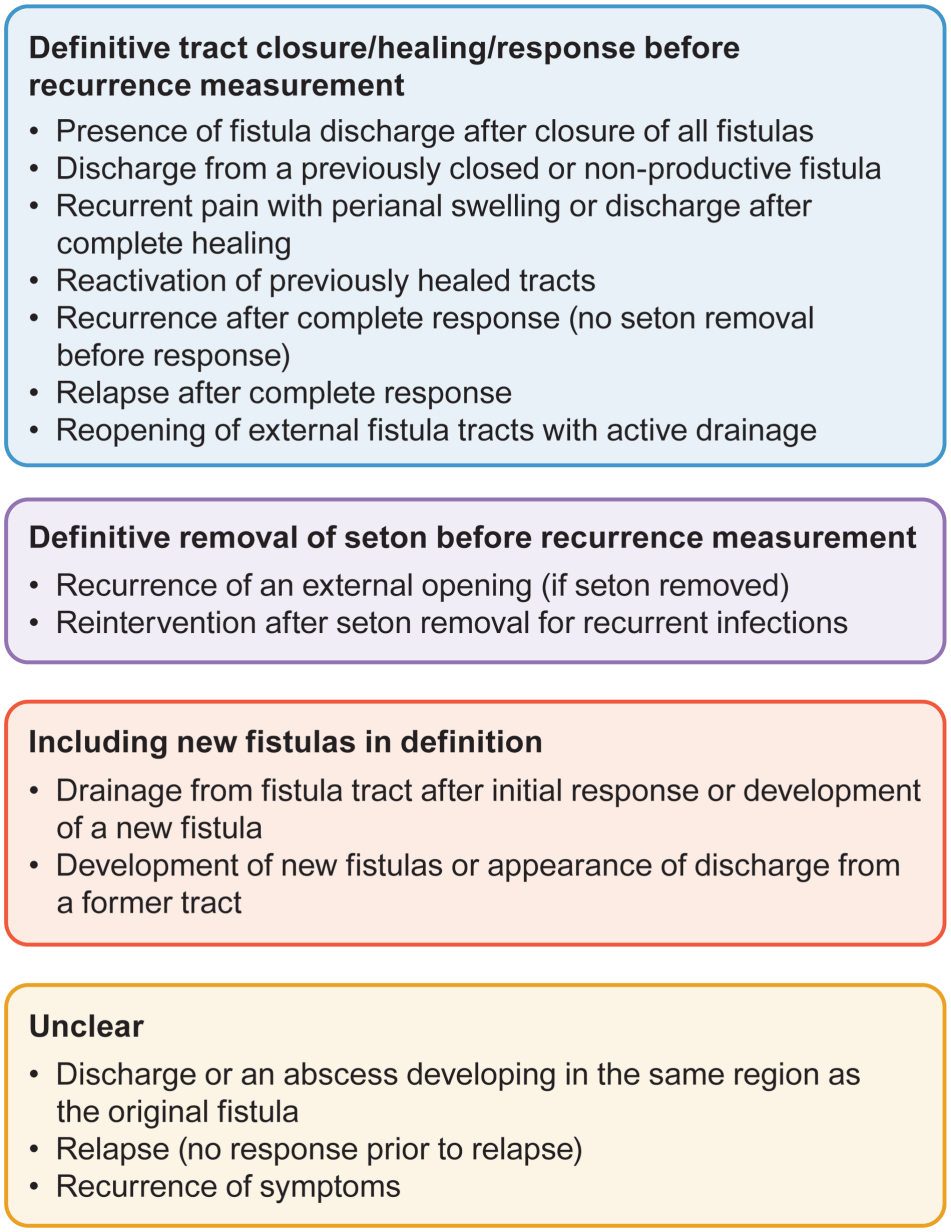
Reported definitions of recurrence in patients with complex Crohn’s perianal fistulas.

**Figure 5.**
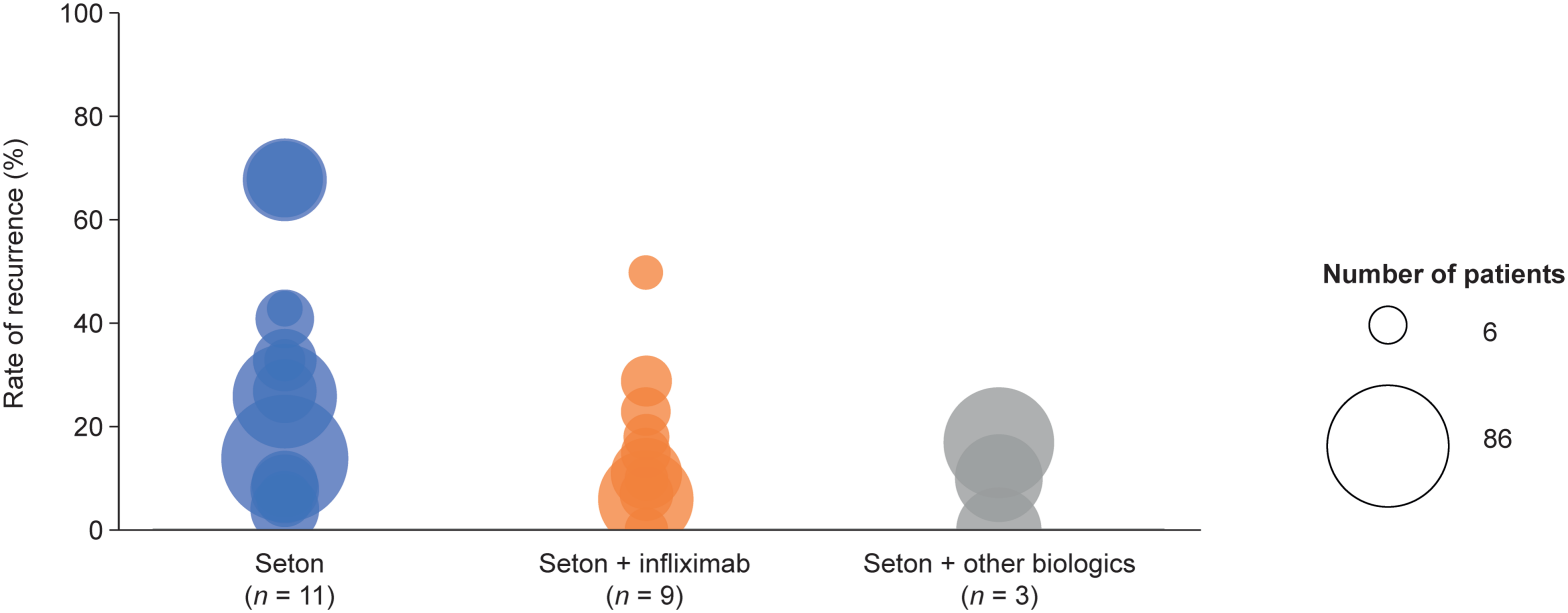
Rates of fistula recurrence. Each circle represents a recurrence rate from a single study, with the size of each circle representing the number of patients investigated in the respective study. The minimum and maximum numbers of patients are shown in the legend. One study (Foo et al^40^) is not included in this figure as it described a specific case with a low patient number and highly variable rates between groups.

In total, 23 studies reported rates of fistula recurrence (Supplementary Table 5). As with studies reporting complete/partial response, definitions for fistula recurrence were varied (Figure 4). Many studies reported rates of recurrence only after complete response had been achieved,^47,48,51,52,56,57,59,70,78,79^ whereas some reported rates of recurrence even in the absence of a complete response.^53,55^ New fistula development was included in the definition of recurrence in 2 studies,^48,49^ and 5 studies did not provide any definition for recurrence.^38,46,58,76,77^ Similar to complete and partial responses, rates of fistula recurrence varied widely, ranging from 4%^55^ to 68%^53^ for patients receiving seton only (*n = *11 studies), 0%^77^ to 50%^59^ for patients receiving seton plus IFX (*n = *9 studies), and 0%^57^ to 17%^52^ for those receiving seton with biologics (*n = *3 studies) (Figure 5). Time to fistula recurrence after seton removal was reported by 11 studies (Supplementary Table 5). Time to fistula recurrence ranged from 3.5 months (mean)^66^ to 61 months (median)^76^ for seton placement only (*n = *4 studies), from 9.5 months (median)^49^ to 19 months (median)^47^ for seton plus IFX (*n = *6), and from 5.5 months (mean)^52^ to 74.8 months (mean)^51^ for seton plus biologics (*n = *2).

#### Rates of fistula-related reintervention

In total, 24 studies assessed rates of fistula-related reintervention (*n = *19 for seton placement only, *n = *4 for seton with IFX, and *n = *3 for seton with biologics) (Supplementary Table 6). As with other clinical outcomes, reintervention rates were varied, regardless of intervention, ranging from 3%^76^ to 71%^32^ for seton only, 0%^66^ to 60%^73^ for seton plus IFX, and 7%^72^ to 61%^61^ for seton plus biologics (Supplementary Table 6). Of the reintervention procedures identified, redrainage and insertion of new or additional setons were most commonly reported, ranging from 8%^74^ to 61%^61^ and 9%^54^ to 71%,^32^ respectively (Supplementary Table 6).

#### Other clinical outcomes

Other clinical outcomes identified in the literature included the rate of new fistula and abscess formation, as well as rates of abscess recurrence (Supplementary Table 6). Overall, 3 studies (all seton only) reported rates of new fistula appearance, which ranged from 9%^58^ to 26%.^54^ The rate of abscess formation was assessed in 6 studies, 4 after seton placement only and 2 after seton placement plus biologics, with rates ranging from 10%^82^ to 47%^42^ and 11%^84^ to 12%,^79^ respectively. Rates of abscess recurrence were reported for seton placement only (*n = *3 studies) and ranged from 7%^76^ to 43%.^75^

#### Patient-reported outcomes

In total, 14 studies assessed PROs (perianal/CD activity, QoL, fecal incontinence, and other functional outcomes). The most commonly used PRO measures were the Perianal Disease Activity Index/Crohn’s Disease Activity Index (PDAI/CDAI, *n = *9 studies), the Inflammatory Bowel Disease Questionnaire (IBDQ, *n = *3^44,69,81^), and the Wexner Fecal Incontinence Score (WFIS, *n = *2^44,55^). PDAI and CDAI were used to assess the response to seton placement alone or in combination with biologics in 8 studies.^29,48,52,61,63,69,74,79^ Of studies comparing baseline scores with scores after treatment (*n = *6 studies^29,48,52,61,74,79^), all found improvements after treatment, and this was independent of the intervention used. Of note, a feasibility study conducted by Stellingwerf et al^74^ investigated the benefit of knotless (SuperSeton) over knotted setons and found that knotless setons significantly reduced fistula discharge and pain, as measured by PDAI, when compared with baseline.

PRO measures (other than PDAI/CDAI) to assess the impact on QoL were reported in 8 studies (2 clinical trials and 6 observational studies).^44,48,55,69,75,80,81,83^ These included the Medical Outcomes Study 36-item Short-Form Health Survey (SF-36) (*n = *1) and a nonvalidated Greek translation of the Cleveland Global QoL (*n = *1).^44^ For other functional outcomes, the most commonly used PRO measure was the IBDQ (*n = *3^44,69,81^) followed by the EuroQol Visual Analogue Scale,^81^ the 5-item International Index of Erectile Function Questionnaire,^44^ the Female Sexual Function Index,^44^ and the Colorectal Functional Outcome Questionnaire.^80^ Of these studies, none compared outcomes between treatment with seton placement only and seton plus IFX or biologics.

#### Clinical outcomes for pediatric and adolescent patients with CPF

Only 4 studies reported outcomes for pediatric or adolescent patients with CPF^30,40,48,60^; all were retrospective chart reviews and had low patient numbers (range 9 patients^30^ to 18 patients^40^). In one study by Rosen et al,^60^ patients receiving endoscopic ultrasound-directed care to monitor healing after seton placement (*n = *10 patients) had a longer time to recurrence of abscess drainage than those monitored by physical examination alone (*n = *4 patients). Two studies described outcomes after combined treatment of seton with IFX. Hukkinen et al^48^ reported that, in adolescent patients receiving seton placement combined with IFX (*n = *13 patients), a complete response was observed in 77% of patients and 15% had a partial response. After 1 year of follow-up, 23% of patients experienced fistula recurrence and by the end of the study, 85% of patients still had a response and 70% were free of perianal symptoms.^48^ Assessing QoL on a scale of 1-7, with 7 being excellent, the following scores were determined after a medium (interquartile range) follow-up time of 2.0 (1.3, 3.8) years after seton placement: physical functioning 7 (7, 7), emotional functioning 7 (6, 7), social functioning 7 (6, 7), and overall QoL 6 (6, 7). Akkelle et al^30^ reported that all 9 patients treated with seton and IFX were free from perianal symptoms and abscess formation after an 18-month follow-up period. Foo et al^40^ reported outcomes after combination treatment with seton and IFX/adalimumab, concluding that the pediatric CDAI may have potential for use as a guide for the timing of seton removal in pediatric patients with CPF.

#### Healthcare resource utilization

Overall, 3 studies reported on HCRU costs for patients with CPF treated with seton only and seton with biologics^36,65,79^; however, most data came from Schwartz et al.^65^

This study reported that all-cause and fistula-related hospitalization costs were lower for patients receiving seton placement prior to biologics when compared with those receiving biologics only (all-cause hospitalization costs: $5514 vs $9711; fistula-related hospitalization costs: $1900 vs $4156). With regard to the main cost drivers associated with CPF treatment, Chaparro et al^36^ reported that pharmacotherapies, particularly biologics, were the main cost driver in complex CPF treatment. Finally, when evaluating the effectiveness of seton plus IFX combination therapy in patients with CPF, Tougeron et al^79^ noted that treatment combination was associated with a reduction in CD-related hospitalizations when comparing baseline and follow-up (0.92 ± 1.13 hospitalizations per patient per year at baseline vs 0.23 ± 0.51 hospitalizations per patient per year during follow-up, *P* = .01).

## Discussion

This SLR was conducted to summarize the available evidence on clinical, patient-reported, and HCRU outcomes associated with seton use for symptomatic relief and treatment of complex CPF. Overall, the results highlight that significant variations exist with respect to reported clinical outcomes after treatment of CPF with seton with or without IFX/other biologics. These observations are likely due to the varied definitions for CPF and associated clinical outcomes, as well as significant variations in study designs and patient characteristics between studies.

Although results were heterogeneous, we did observe an overall trend of improved outcomes in patients who receive seton with IFX or other biologics when compared with those receiving seton treatment only. The few studies that directly compared patients treated with seton alone or seton + IFX/biologics reported an improved clinical response or significantly longer time to fistula recurrence in patients who received seton with IFX/biologics compared to those treated with seton alone. Further studies using standardized study protocols and homogeneous patient populations would be required to confirm this trend.

The significant heterogeneity in clinical outcome definitions identified with respect to seton use in CPF highlights a need for standardization across all intervention modalities in this disease setting. In a recent literature review by Sahnan et al,^85^ 295 different CPF-related clinical outcomes were identified, and this variability is consistent with observations in our study. Such heterogeneity clearly impedes analysis of treatment effectiveness across studies. In their study, Sahnan et al^85^ proposed a core set of outcomes for CPF, which aimed to provide a standardized framework for the use of patient-reported, clinician-reported, and imaging outcomes in studies assessing patients with CPF. Currently, definitions such as the Parks’ classification^86^ or AGA definition^13^ of perianal fistulas are widely used; however, as highlighted in this study, the application of these definitions is inconsistent between studies. Recent work by Geldof et al^15^ proposed a new classification of CPF, which categorizes patients according to 4 classes (class 1: minimal disease; class 2: chronic symptomatic fistulas; class 3: severe disease with exhausted perineum and adverse features; and class 4: perineal symptoms after proctectomy), with stratification guided by severity, disease outcome, synchronization of patient and clinician goals in decision-making, and identification of indications for curative fistula treatment, diverting ostomy, and proctectomy. Importantly, each patient category is paired with a treatment strategy and a description of clinical trial suitability.^15^ This new classification system may reduce variability in both CPF definitions and patient populations across studies and represents a significant step towards the standardization of outcomes in future research. Using a standardized classification system may allow for more consistent use of setons in patients with CPF; however, understanding the role of setons in the CPF treatment algorithm and the optimal time for their removal remains a matter for clarification. The timing of seton removal may be affected by both clinical and patient-related factors. In this study, we identified a paucity of clinical data with respect to the optimal timing of seton removal in patients with CPF, with wide variation in the duration of seton placement in patients regardless of intervention strategy. Only one study identified an association between clinical benefit and timing of seton removal (<34 weeks of seton drainage was associated with sustained fistula closure in patients receiving seton placement and IFX^35^); however, other studies indicated that some patients prefer to delay seton removal, even after cessation of drainage and alleviation of anal pain, owing to the anxiety associated with potential abscess recurrence. The absence of clear data on the optimal duration of seton placement and/or criteria for seton removal is a gap identified by this SLR. Future clinical studies are required if this important clinical question is to be addressed and clear recommendations on the duration of seton placement and timing of removal are to be formulated.

In the current study, few definitions of complete response/remission, partial response, or recurrence referred to radiological metrics, although some studies did use radiological techniques to detect and characterize fistulas as well as to determine treatment outcomes. A lack of radiological assessment can lead to a lack of clinical understanding with respect to disease status and subsequent suboptimal treatment of the patient. However, as with clinical outcome definitions, there are also significant variations regarding definitions for radiological outcomes in CPF. A recent systematic review and meta-analysis by Lee et al^87^ identified a need for consensus regarding the definition of improvement, as determined by MRI, in patients with CPF.

The present study also identified several significant gaps in the literature. Only 4 studies were identified that reported clinical outcomes in pediatric/adolescent patients with complex CPF. In addition, few studies reported outcomes relating to the optimal timing of seton removal, HCRU or PROs, and such evidence gaps may be restricting the development of optimal disease management and treatment strategies across different patient populations with CPF. Furthermore, we only identified 6 RCTs, which is in accordance with a previous study that noted data from clinical trials (where treatment of CPF was a primary objective) were limited.^20^

It is therefore clear that standardized approaches with regard to clinical and radiological outcome definitions, as well as use of PRO measures, are required in this disease area to permit cross-study comparisons and to help inform effective patient treatment algorithms in CPF. Further research into CPF-related HCRU and pediatric/adolescent CPF populations is required, alongside an increase in the number of RCTs where CPF treatment is the focus to enhance the evidence base and thus inform future treatment algorithms.

### Strengths of This SLR

This SLR was conducted according to the guidelines of the Cochrane Collaboration, CRD, and NICE. It represents a comprehensive review of the current state of the literature, because most studies (*n = *40) were published between 2010 and 2021. Although new data may have become available since this SLR was completed, the data presented in this study are relevant and represent the key studies that provide a foundation for informing future evidence-generation needs.

### Limitations of This SLR

The results of this SLR should be interpreted within the context of several limitations. Owing to the observational nature of most of the studies (*n = *50), overall quality assessment results revealed a moderate-to-serious risk of bias in the findings. As discussed, heterogeneity in patient populations, study designs, clinical outcome, and CPF definitions precluded the comparison of results between studies. Interpretation of results was further impeded by most studies having at least a moderate risk of bias and a lack of RCTs (*n = *6), meaning that the evidence base for treatment outcomes after seton was limited with regard to scientific quality. In addition, although studies often reported the use of concomitant medications by patients, it was not possible to determine how the use of concomitant medications (eg, steroids and antibiotics) affected clinical outcomes associated with seton use. Finally, studies directly comparing the impact of seton use with the outcome of other CPF-related procedures were not available. It is therefore not possible to draw inferences regarding optimal seton treatment paradigms.

## Conclusions

This SLR highlights a dearth of clarity and high-quality studies addressing the optimal use of setons in patients with complex CPF. There was seemingly a trend towards improved clinical and patient outcomes when seton treatment was combined with IFX or biologics compared with seton placement alone; however, the heterogeneous nature of the outcomes identified precluded cross-study comparisons. Although there was a degree of consistency among the PRO measures used, only a quarter of studies reported PRO data. Data were also limited for HCRU and radiological outcomes, as well as for the impact of seton placement on the clinical outcomes of pediatric/adolescent patients with complex CPF, representing a significant gap in the literature. There is a clear need for internationally recognized, standardized definitions for CPF and clinical outcomes, alongside standardized CPF study designs, as well as for an increase in studies in certain areas to permit cross-study comparability. This will elicit clarity on the impact of seton use in patients with complex CPF and inform optimal seton-based treatment strategies in this patient population.

## Supplementary Data

Supplementary data is available at *Inflammatory Bowel Diseases* online.

izae186_suppl_Supplementary_Tables

## Data Availability

The data sets/references used and/or analyzed during the current study are available from the corresponding author upon reasonable request.
