# Supplementary material for: Impact of Seton Use on Clinical, Patient-Reported, and Healthcare Resource Utilization Outcomes in Complex Crohn’s Perianal Fistulas: A Systematic Literature Review
Source: Inflamm Bowel Dis. 2024 Sep 19;31(6):1556–66. doi: 10.1093/ibd/izae186 (PMC12166306; doi:10.1093/ibd/izae186)
Supplement: izae186_suppl_Supplementary_Tables [file izae186_suppl_supplementary_tables.docx]

# Supplementary tables

## Supplementary Table 1. PICOS-T criteria for the inclusion and exclusion criteria of studies

| PICOS-T | Inclusion criteria | Exclusion criteria |
| --- | --- | --- |
| Population | CD diagnosis  Complex CPF | Non-human |
| Interventions | Seton placement as palliative treatment | Seton placement as preparatory procedure for surgeries |
| Comparators | No restrictions | N/A |
| Outcomes | Clinical endpoints:   - Any short- or long-term outcomes, including healing rate or cessation of drainage, recurrence rate, success rate, response rate, fistula closure, clinical remission, clinical response - PROs [pain, incontinence, QoL, emotional impact, RFIS, Wexner Scale of Incontinence, EQ5D, SF-36, WPAI, IBDQ, sexual activity impairment etc.]   Clinical outcomes of post-seton procedures:   - Focus on seton as palliative treatment - Stratify based on seton vs no seton   HCRU and costs associated with seton placement in patients with complex CPF:   - Inpatient/outpatient, hospitalizations, and associated costs, including emergency room visits, laboratory and diagnostics tests, direct/indirect costs - Post-surgical complications post-seton placement   Patient characterization:   - Demographics - Clinical characteristics of fistula [e.g. single/branched] and CD [e.g. CD duration, disease location, CDAI score] - Medical and surgical history prior to seton placement as palliative treatment | All publications that do not cover at least one of the outcomes |
| Study design | Any clinical trials [including RCTs, single-arm trials]  Observational studies and case reports with *n*≥ 5 patients  SLRs, meta-analyses, review articles, guidelines [included only for cross-reference check] | Animal/*in vitro* studies  Case reports with *n*< 5 patients |
| Time | 01/01/1980 to date | Any publication before 1980 |

CD, Crohn’s disease; CDAI, Crohn’s Disease Activity Index; CPF, Crohn’s perianal fistula; EQ5D, EuroQol 5 Dimension; HCRU, healthcare resource utilization; IBDQ, Inflammatory Bowel Disease Questionnaire; N/A, not applicable; PICOS-T, population, intervention, comparator, study design, time; PRO, patient-reported outcome; QoL, quality of life; RCT, randomized controlled trial; RFIS, Revised Fecal Incontinence Scale; SF-36, 36-Item Short-Form Health Survey; SLR, systematic literature review; WPAI, Work Productivity and Activity Impairment.

## Supplementary Table 2. Study characteristics: observational studies

| **Intervention** | **Study** | **Ref.** | **FT or AB** | **Country** | **Study type, data source** | **Number of patients^a^** | **Follow-up,^b^ range [months]** | **DOI** |
| --- | --- | --- | --- | --- | --- | --- | --- | --- |
| Seton | Banayan *et al*. 2016 | 30 | AB | USA | Retrospective, medical chart review | 41 | 35, 8–69 | https://dx.doi.org/10.1111/codi.13445 |
| Seton | Chaparro *et al*. 2013 | 34 | FT | Spain | Cross-sectional and retrospective | 97 | 4.2 ± 1.5 years | http://dx.doi.org/10.1007/s10620-013-2830-7 |
| Seton | Faucheron *et al*. 1996 | 36 | FT | France | Retrospective, medical chart review | 41 | 47, 5–104 | https://dx.doi.org/10.1007/BF02068077 |
| Seton | Felton *et al*. 2020 | 37 | AB | USA | Retrospective, database | 76 | - | https://dx.doi.org/10.1097/DCR.0000000000001712 |
| Seton | Galis-Rozen *et al*. 2010 | 40 | FT | Israel | Retrospective, clinical records review | 17 [77] | 24, 6–48 | https://dx.doi.org/10.1111/j.1463-1318.2009.01796.x |
| Seton | Gklavas *et al*. 2021 | 42 | FT | Greece | Cross-sectional, survey | 66 [95] | 36 | https://dx.doi.org/10.1007/s11605-021-04987-2 |
| Seton | Koganei *et al*. 1995 | 48 | FT | Japan | Retrospective, medical chart review | 13 | 12.1 [mean], 6.0–18.2 | https://dx.doi.org/10.1007/BF00309382 |
| Seton | Motamedi *et al*. 2021 | 51 | FT | Canada | Retrospective, medical chart review | 77 [177] | 31, 15–54 [IQR] | https://dx.doi.org/10.1111/codi.15771 |
| Seton | Mujukian *et al*. 2020 | 52 | FT | USA | Retrospective, cohort study | 23 | 58, 12–133 | https://dx.doi.org/10.1177/0003134820964462 |
| Seton | Papaconstantinou *et al*. 2017 | 53 | FT | Greece | Retrospective, medical chart review | 25 [59] | 1.6 ± 1.1 years [mean ± SD] | https://dx.doi.org/10.1177/1457496916665763 |
| Seton | Park *et al*. 2021 | 54 | FT | South Korea | Retrospective, medical chart review | 141 [153] | 28.8 | https://dx.doi.org/10.1016/j.asjsur.2021.03.013 |
| Seton | Reddy *et al*. 2016 | 56 | AB | USA | Retrospective, medical chart review | 57 | - | http://dx.doi.org/10.1097/01.dcr.0000482708.50838.af |
| Seton | Rosen *et al*. 2010 | 58 | FT | USA | Retrospective, medical chart review | 14 | ≥1 year | https://dx.doi.org/10.1002/ibd.21067 |
| Seton | Schaad *et al*. 2021 | 60 | AB | Switzerland | Retrospective, database analysis [of a prospective cohort] | 98 [365] | 7.5, 0–12.6^c^ | https://dx.doi.org/10.1093/bjs/znab202.008 |
| Seton | Scott *et al*. 1996 | 65 | FT | UK | Retrospective, hospital, and operating records, survey | 27 [59] | 12–35^c^ | https://dx.doi.org/10.1007/BF02054696 |
| Seton | Sebastian *et al*. 2018 | 22 | FT | Europe, Israel | Retrospective, cohort study | 136 [253] | ≥12 | https://dx.doi.org/10.1111/apt.14969 |
| Seton | Shinozaki *et al*. 2002 | 67 | FT | Japan | Retrospective, medical chart review | 39 | 21.4 [mean] | https://dx.doi.org/10.1007/s005350200097 |
| Seton | Sibio *et al*. 2018 | 68 | FT | Italy | Prospective | 32 [61] | ≥12 | https://dx.doi.org/10.1155/2018/5249087 |
| Seton | Sugita *et al*. 1995 | 72 | FT | Japan | Retrospective | 21 [119] | 16, 1–37 | - |
| Seton | Takesue *et al*. 2002 | 73 | FT | Japan | - | 32 | 62, 25–133 | https://dx.doi.org/10.1007/s005350200153 |
| Seton | Van Koperen *et al*. 2009 | 77 | FT | Netherlands | Retrospective | 24 [61] | 79, 13–140 | https://dx.doi.org/10.1002/bjs.6608 |
| Seton | White *et al*. 1990 | 79 | FT | USA | Retrospective | 10 | 2 months to 7 years | https://dx.doi.org/10.1007/BF02052212 |
| Seton | Williams *et al*. 1991 | 80 | FT | USA | - | 23 [74] | 24, 4–60 | https://dx.doi.org/10.1002/bjs.1800781004 |
| Seton + ADA | Solina *et al*. 2016 | 69 | AB | Italy | Prospective | 58 | - | https://doi.org/10.1093/ecco-jcc/jjw019.580 |
| Seton + anti-TNF | Hafi *et al*. 2017 | 43 | AB | Tunisia | Retrospective | 49 | >1 year | https://dx.doi.org/10.1177/2050640617725676 |
| Seton ± anti-TNF [IFX/ADA] | El-Gazzaz *et al*. 2012 | 35 | FT | USA | Retrospective, database/medical chart review | 90 [218] | 3.32 ± 2.99 years [mean ± SD]^c^ | https://dx.doi.org/10.1111/j.1463-1318.2012.02944.x |
| Seton + anti-TNF [IFX/ADA] | Foo *et al*. 2021 | 38 | AB | Australia | Retrospective, review of patient files | 18 | - | https://dx.doi.org/10.1097/MPG.0000000000003177 |
| Seton + anti-TNF [IFX/ADA] | Kotze *et al*. 2014 | 49 | FT | Brazil | Retrospective, medical chart review | 78 | 48.2 [mean], 2–228 | https://dx.doi.org/10.1590/S0004-28032014000400004 |
| Seton + anti-TNF [IFX/ADA] | Lin *et al*. 2016 | 50 | FT | China | Retrospective, medical chart review | 65 | 25.3 [mean], 3–84 | https://dx.doi.org/ 10.3892/etm.2016.3552 |
| Seton + anti-TNF [IFX/ADA] | Park *et al*. 2021 | 55 | FT | South Korea | Retrospective, medical chart review | 39 [69] | 46, 30–52.5 | https://dx.doi.org/10.1186/s13287-021-02484-6 |
| Seton + anti-TNF [IFX/ADA] | Yardimci *et al*. 2016 | 81 | FT | Turkey | Retrospective, medical chart review | 27 | 17, 6–32 | https://doi.org/10.5472/MMJoa.2902.03 |
| Seton + anti-TNF [IFX/ADA/CER] | Schwartz *et al*. 2017 | 63 | FT | USA | Retrospective, claims data | 326 [1845] | 6 | https://dx.doi.org/10.1097/MIB.0000000000001243 |
| Seton + IFX/ADA/UST | Saigusa *et al*. 2018 | 59 | FT | Japan | - | 18 | 4.0 [mean], 0.4–6.8 years | https://dx.doi.org/10.23922/jarc.2017-044 |
| Seton + IFX | Akkelle *et al*. 2021 | 28 | FT | Turkey | Retrospective, medical chart review | 9 [50] | 38 ± 21 [mean ± SD] | https://dx.doi.org/10.5152/tjg.2021.191034 |
| Seton + IFX | Antakia *et al*. 2010 | 29 | AB | UK | Retrospective | 30 | 20 | http://dx.doi.org/10.1111/j.1463-1318.2010.02407.x |
| Seton + IFX | Bouguen *et al*. 2013 | 33 | FT | France | Retrospective, medical chart review | 97 [156] | 250, 124–381 [IQR] weeks^c^ | https://dx.doi.org/10.1016/j.cgh.2012.12.042 |
| Seton ± IFX | Gaertner *et al*. 2007 | 39 | FT | USA | Retrospective, medical chart review | 112 | 30 [mean], 6–216 months^c^ | https://dx.doi.org/ 10.1007/s10350-007-9077-3 |
| Seton + IFX | Gao *et al*. 2013 | 41 | AB | China | Retrospective | 14 | - | https://dx.doi.org/ http://dx.doi.org/10.1111/jgh.12363_2 |
| Seton + IFX | Higashi *et al*. 2009 | 44 | FT | Japan | - | 86 | 68.8 [mean], 12–184 | - |
| Seton + IFX | Hotokezaka *et al*. 2011 | 45 | FT | Japan | - | 20 | 31.8 ± 4.2 [mean ± SD] | https://dx.doi.org/10.5754/hge09586 |
| Seton + IFX | Hukkinen *et al*. 2014 | 46 | FT | Finland | Retrospective, medical record review | 13 | 2.0, 1.3–3.8 [IQR] years after SP | https://dx.doi.org/10.1016/j.crohns.2014.01.001 |
| Seton + IFX | Jeon *et al*. 2019 | 47 | FT | South Korea | Retrospective, medical record review | 76 | 21.0 ± 11.6 [mean ± SD] | https://dx.doi.org/10.3393/ac.2018.11.23.1 |
| Seton + IFX | Regueiro *et al*. 2003 | 57 | FT | USA | Retrospective, medical record review | 9 [32] | ≥3 months after 3rd dose of IFX | https://doi.org/10.1097/00054725-200303000-00003 |
| Seton + IFX | Schwartz *et al*. 2005 | 62 | FT | USA | Retrospective | 14 [21] | 68, 35–101 weeks^c^ | https://dx.doi.org/ 10.1097/01.mib.0000172811.57242.18 |
| Seton ± IFX | Sciaudone *et al*. 2010 | 64 | FT | Italy | Prospective | 24 [35] | 18.8, 8–38^c^ | - |
| Seton + IFX | Talbot *et al*. 2005 | 74 | FT | UK | Prospective | 21 | 20, 12–52 | https://dx.doi.org/ 10.1111/j.1463-1318.2004.00749.x |
| Seton + IFX | Tanaka *et al*. 2010 | 75 | FT | Japan | - | 14 | 12.1 [mean], 3–33 | - |
| Seton + IFX | Tougeron *et al*. 2009 | 76 | FT | France | Retrospective, medical chart review | 26 | 4.9 ± 9.6 [mean ± SD] | https://dx.doi.org/10.1007/s10620-008-0545-y |
| Seton + UST | Biron *et al*. 2019 | 31 | AB | France | - | 88 [207] | 66 [mean] weeks^c^ | https://doi.org/10.1093/ecco-jcc/jjy222.108 |
| Seton + VED | Biron *et al*. 2019 | 32 | AB | France | - | 61 [151] | 86 [mean] weeks^c^ | https://doi.org/10.1093/ecco-jcc/jjy222.112 |

^a^Relevant subgroup [overall patient number]; reasons for patient group of interest being smaller: not all patients had CD, not all patients received setons, additional interventions not of interest investigated.

^b^Median, range in months [unless stated otherwise].

^c^Follow-up period available only for the overall patient group.

AB, abstract; ADA, adalimumab; CER, certolizumab; FT, full-text; IFX, infliximab; IQR, interquartile range; SD, standard deviation; SP, seton placement; TNF, tumor necrosis factor; UST, ustekinumab; VED, vedolizumab.

## Supplementary Table 3. Study characteristics: randomized controlled trials

| **Intervention** | **Study** | **Ref.** | **FT or AB** | **Country** | **Study type, data source** | **Number of patients^a^** | **FU [median, range in months; unless stated otherwise]** | **DOI** |
| --- | --- | --- | --- | --- | --- | --- | --- | --- |
| Seton | Senejoux *et al.* 2016 | 66 | FT | France, Belgium | RCT, crossover after primary endpoint | 52 [106] | 1 year | https://dx.doi.org/10.1093/ecco-jcc/jjv162 |
| Seton | Stellingwerf *et al.* 2020 | 71 | FT | Netherlands | Single-arm trial, survey | 41 [60] | 3 | https://dx.doi.org/10.1038/s41598-020-73737-2 |
| Seton + ADA | Abramowitz *et al.* 2019 | 27 | AB | France | Randomized, prospective multicentric trial | 31 [64] | - | https://dx.doi.org/10.1093/ecco-jcc/jjy222.684 |
| Seton ± anti-TNF | Wasmann *et al.* 2020 | 78 | FT | Netherlands, Belgium, Spain, Italy | RCT and prospective registry data | RCT: 30 [44]; registry: 41 [50] | 6–18 | https://dx.doi.org/10.1093/ecco-jcc/jjaa004 |
| Seton + CER | Schwartz *et al.* 2015 | 61 | AB | USA | Randomized, prospective multicenter trial | - [21] | - | https://dx.doi.org/10.1016/S0016-5085(15)30903-3 |
| Seton + IFX | Spradlin *et al.* 2008 | 70 | FT | USA | RCT, partly blinded | 5 [10] | 54 weeks | https://dx.doi.org/10.1111/j.1572-0241.2008.02063.x |

^a^Relevant subgroup [overall patient number]; reasons for patient group of interest being smaller: not all patients had CD, not all patients received setons, additional interventions not of interest investigated.

AB, abstract; ADA, adalimumab; CER, certolizumab; IFX, infliximab; FT, full-text; FU, follow-up; RCT, randomized controlled trial; TNF, tumor necrosis factor.

## Supplementary Table 4. Studies reporting complete/partial response

| **Intervention** | **Study** | **Fistula type** | **Ref.** | **Seton duration in months^a^** | **COMPLETE RESPONSE** | | | | **PARTIAL RESPONSE** | | | |
| --- | --- | --- | --- | --- | --- | --- | --- | --- | --- | --- | --- | --- |
|  |  |  |  |  | **Def.** | **Def. from paper** | **Pts [*n*]** | **Rate [%]** | **Def.** | **Def. from paper** | **Pts [*n*]** | **Rate [%]** |
| Seton | El-Gazzaz *et al.* 2012 | NR | 35 | NR | NR | NR | NR | NR | Closure or improvement | Healed or improved fistula [healed: closed fistula opening with no associated symptoms for >2 weeks; improved: minimal symptoms/drainage, biological dependence] | 37 | ~20^c^ |
| Seton | Faucheron *et al.* 1996 | Complex | 36 | 12 [mean], 4–60 | Not defined | Remission | 18 | 61^b^ | NR | NR | NR | NR |
| Seton | Gaertner *et al.* 2007 | Complex | 39 | NR | Closure | Fistula healing: no clinical evidence of a fistula tract | NR | ~17^c^ | NR | NR | NR | NR |
| Seton | Galis-Rozen *et al.* 2010 | Complex | 40 | ≥FU | NR | NR | NR | NR | Improvement | Significant clinical improvement | 17 | 59 |
| Seton | Higashi *et al.* 2009 | Mixed | 44 | NR | Not defined | Remission | 86 | 26^b^ | NR | NR | NR | NR |
| Seton | Koganei *et al.* 1995 | Complex | 48 | Long-term | NR | NR | NR | NR | Improvement | Overall good result: improvement or disappearance of pyrexia, perianal pain, discharge, induration, and tenderness | 13 | 77 |
| Seton | Motamedi *et al.* 2021 | Mixed | 51 | 10, 6–17 [IQR] | NR | NR | NR | NR | Cessation of drainage or improvement | Healed [fistula closed with no drainage] or controlled [minimal drainage or pain] fistula, initial seton removed | 43 | 49 |
| Seton | Mujukian *et al.* 2020 | Mixed | 52 | ≥12 | NR | NR | NR | NR | Improvement | Clinical response: lack of induration, pain, swelling, abscess recurrence, or unintended dislodgement | 19 | 16 |
| Seton | Park *et al.* 2021 | Complex | 54 | 14, 1.4-41.4 | Closure | Closure rate: complete healing of perianal wound, absence of fluid from fistula after seton removal | 112 | 15^b^ | NR | NR | NR | NR |
| Seton | Park *et al.* 2021 | Mixed | 54 | 14, 1.4–41.4 | Closure | Closure rate: complete healing of perianal wound, absence of fluid from fistula after seton removal | 141 | 18^b^ | NR | NR | NR | NR |
| Seton | Schaad *et al.* 2021 | NR | 60 | NR | Closure | Fistula closure | 98 | 75^e^ | NR | NR | NR | NR |
| Seton | Sciaudone *et al.* 2010 | Complex | 64 | NR | Closure | Complete response: closure of all external openings, cessation of drainage for >3 months | 10 | 70^b^ | Improvement | Partial response: reduction in the size, number, drainage, discomfort | 10 | 20 |
| Seton | Scott *et al.* 1996 | Mixed | 65 | 14, 2–22 | NR | NR | NR | NR | Improvement | Successful outcome: satisfaction of patient and surgeon with result of overall Tx | 27 | 85 |
| Seton | Senejoux *et al.* 2016 | Complex | 66 | >1 month | Closure | Remission: cessation of drainage and healing of the external orifice, remission of clinical signs and symptoms | 13 | 15^b^ | NR | NR | NR | NR |
| Seton | Senejoux *et al.* 2016 | Mixed | 66 | >1 month | Closure | Remission = cessation of drainage and healing of the external orifice, remission of clinical signs and symptoms | 52 | 23^b^ | NR | NR | NR | NR |
| Seton | Shinozaki *et al.* 2002 | Mixed | 67 | Long-term | NR | NR | NR | NR | Cessation of drainage | Healed anal fistula: no discharge noted for >1 month after removal of all setons | 39 | 67 |
| Seton | Sugita *et al.* 1995 | Mixed | 72 | >3 | Cessation of drainage | All setons removed and no recurrent fistulous abscess had developed | 17 | 47^b,d^ | Not defined | Good result | 21 | 81 |
| Seton | Takesue *et al.* 2002 | Mixed | 73 | Simple removal [*n =*9]: 11, 6–18; with post-seton procedure [*n =*11]: 9, 6–13 | NR | NR | NR | NR | Cessation of drainage | Thin, non-productive fistula or quiescent, all external wounds healed, leaving only the rigid, fibrotic single-tract fistula containing the seton | 32 | 63 |
| Seton | White *et al.* 1990 | Complex | 79 | 6, 2–88 | NR | NR | NR | NR | Improvement | Excellent palliation with decreased pain, resolution of sepsis, maintenance of continence, no creation of surgical wound | 10 | 100 |
| Seton | Williams *et al.* 1991 | Complex | 80 | Long-term | Closure | Good result: healed wounds | 23 | 13^b^ | Cessation of drainage | Good result: fistula tract remained quiescent without abscess but did not close | 23 | 35 |
| Seton + ADA | Abramowitz *et al.* 2019 | Complex | 27 | NR | Closure | Fistula closure | 48 | 52 | NR | NR | NR | NR |
| Seton + ADA | Abramowitz *et al.* 2019 | Mixed | 27 | NR | Closure | Fistula closure | 64 | 59 | NR | NR | NR | NR |
| Seton + ADA | Solina *et al.* 2016 | Complex | 69 | NR | Closure | Clinical remission: closure of all fistulas that were draining at baseline for >2 consecutive visits [at least 4 weeks] | 58 | 34 | Closure or improvement | Clinical benefit: either clinical remission or clinical improvement 50% decrease from baseline in number of draining fistulas for >2 consecutive visits [at least 4 weeks] | 58 | 74 |
| Seton + anti-TNF | Hafi *et al*. 2017 | Complex | 43 | NR | Not defined | Clinical remission | 49 | 53 | Not defined | Partial clinical response | 49 | 31 |
| Seton + anti-TNF [IFX/ADA] | El-Gazzaz *et al.* 2012 | NR | 35 | NR | NR | NR | NR | NR | Closure or improvement | Healed or improved fistula [healed: closed fistula opening with no associated symptoms for >2 weeks; improved: minimal symptoms/drainage, biological dependence] | 53 | ~65^c^ |
| Seton + anti-TNF [IFX/ADA] | Kotze *et al.* 2014 | Mixed | 49 | 6, 1–36 | Closure | Complete perianal remission: complete healed perineum, without active fistulas, drainage, or setons | 78 | 42^b^ | Cessation of drainage | Partial response: absence of fistula drainage but persistence of seton as a patient’s option | 78 | 10 |
| Seton + anti-TNF [IFX/ADA] | Lin *et al.* 2016 | Mixed | 50 | NR | Closure | Complete fistula closure, where improvement lasted throughout FU without recurrence; fistula closed completely without exudate or pain | 65 | 52 | Improvement | External orifice exudation, where fistula was not completely closed, and fluid exuded from an external orifice | 65 | 15 |
| Seton + anti-TNF [IFX/ADA] | Park *et al.* 2021 | Mixed | 55 | NR | Closure | Cumulative fistula closure: absence of discharge, swelling, or pain | 39 | 23 | NR | NR | NR | NR |
| Seton + CER | Schwartz *et al.* 2015 | Mixed | 61 | NR | Closure | Fistula healing | 21 | 50 | NR | NR | NR | NR |
| Seton + IFX/ADA/UST | Saigusa *et al.* 2018 | Mixed | 59 | 2.6 [mean], 1.4–5.7 years | Closure | Healed [fistula was not palpable, with mucosal healing of primary opening and closure of secondary opening after seton removal] | 18 | 39^b^ | Cessation of drainage | Fistula no longer drained despite gentle finger compression, setons removed successfully | 18 | 56 |
| Seton + anti-TNF [IFX/ADA] | Yardimci *et al.* 2016 | Mixed | 81 | NR | NR | NR | NR | NR | Cessation of drainage | Absence of drainage from all fistulas despite gentle finger compression with or without cicatrization of external fistula orifice | 27 | 63 |
| Seton + IFX | Akkelle *et al.* 2021 | Mixed | 28 | 6–12 | Not defined | Clinical remission | 9 | 100^b,h^ | NR | NR | NR | NR |
| Seton + IFX | Antakia *et al.* 2010 | NR | 29 | NR | Not defined | Complete response | 29 | 23 | Not defined | Partial response | 29 | 50 |
| Seton + IFX | Bouguen *et al.* 2013 | Mixed | 33 | 33, 14–64 [IQR] weeks | Closure | Sustained perianal fistula closure | 97 | ~65^b,c^ | NR | NR | NR | NR |
| Seton + IFX | Gaertner *et al.* 2007 | Complex | 39 | NR | Closure | Fistula healing: no clinical evidence of a fistula tract | NR | ~42^c^ | NR | NR | NR | NR |
| Seton + IFX | Gao *et al.* 2013 | NR | 41 | NR | Not defined | Complete response | 14 | 64 | Improvement | Decreased drainage, setons not removed | 14 | 7 |
| Seton + IFX | Higashi *et al.* 2009 | Mixed | 44 | NR | Not defined | Good response: required no additional Tx | 19 | 42 | Improvement | Intermediate response: required FU care other than surgical Tx | 19 | 26 |
| Seton + IFX | Hotokezaka *et al.* 2011 | Mixed | 45 | 20.8 ± 4.6 [mean ± SD] | Cessation of drainage | Complete response: no discharge and no anal pain was observed with gentle finger compression | 20 | 75^k^ | Improvement | Partial response: reduction in size, number and in drainage volume but where discharge was observed with finger compression | 20 | 25 |
| Seton + IFX | Hukkinen *et al.* 2014 | Mixed | 46 | 8, 5–10.5 [IQR] | Cessation of drainage | Complete remission: complete closure of tract with cessation of drainage from the external opening | 13 | 77 | Improvement | Partial response: reduction in the size, number, or drainage of fistulas | 13 | 15 |
| Seton + IFX^i^ | Jeon *et al.* 2019 | Mixed | 47 | NR | Closure | Complete remission: complete closure of tract, cessation of drainage from external opening | 49 | 65 | Improvement | Partial response: reduction of discharge and/or reduction in size and number of fistulas | 49 | 35 |
| Seton + IFX^j^ | Jeon *et al.* 2019 | Mixed | 47 | NR | Closure | Complete remission: complete closure of tract, cessation of drainage from external opening | 27 | 63 | Improvement | Partial response: reduction of discharge and/or reduction in size and number of fistulas | 27 | 37 |
| Seton + IFX | Regueiro *et al.* 2003 | Complex | 57 | Up to 2–4 weeks after 2nd IFX infusion | Closure | Fistula closure within 3 months of 3rd induction dose of IFX | 6 | 100^b^ | NR | NR | NR | NR |
| Seton + IFX | Regueiro *et al.* 2003 | Mixed | 57 | Up to 2–4 weeks after 2nd IFX infusion | Closure | Fistula closure within 3 months of 3rd induction dose of IFX | 9 | 100^b^ | NR | NR | NR | NR |
| Seton + IFX | Schwartz *et al.* 2005 | Complex | 62 | NR | NR | NR | NR | NR | Cessation of drainage | Inability to express purulent material from fistula with application of gentle pressure to tract | 8/7^g^ | 100/43^g^ |
| Seton + IFX | Schwartz *et al.* 2005 | Complex | 62 | NR | NR | NR | NR | NR | Cessation of drainage [EUS] | Fistula was considered inactive at EUS when the hypoechoic echotexture of tract became more heterogeneous and closely approximated the seton | 8/7^g^ | 75/14^g^ |
| Seton + IFX | Sciaudone *et al.* 2010 | Complex | 64 | - | Closure | Complete response: closure of all external openings, cessation of drainage for >3 months | 14 | 79^b^ | Improvement | Partial response: reduction in the size, number, drainage, discomfort | 14 | 14 |
| Seton + IFX | Spradlin *et al.* 2008 | Complex | 70 | 278.5 days | Closure | Fistula closed if investigator cannot express purulent material from the fistula with the application of gentle pressure to the tract | 5 | 80^b^ | NR | NR | NR | NR |
| Seton + IFX | Talbot *et al.* 2005 | Complex | 74 | NR | Closure | Complete response: complete closure of fistula with no further drainage on gentle finger pressure | 21 | 47^b^ | Improvement | Partial response: reduction in size or number or drainage or improvement of pain | 21 | 52 |
| Seton + IFX | Tanaka *et al.* 2010 | NR | 75 | Until 3rd IFX infusions: 54%; 4th: 9%; 5th: 18%; 8th: 18% | Closure | Complete response: no discharge from fistulas, all drains removed, all fistulas closed | 14 | 79^b^ | Improvement | Partial response: partial removal of the drains | 14 | 14 |
| Seton + IFX | Tougeron *et al.* 2009 | Complex | 76 | NR | NR | NR | NR | NR | Cessation of drainage | Absence of drainage from all fistulas with or without cicatrization of the external fistula orifice |  | 44^f^ |
| Seton + IFX | Tougeron *et al.* 2009 | Mixed | 76 | NR | NR | NR | NR | NR | Cessation of drainage | Absence of drainage from all fistulas with or without cicatrization of the external fistula orifice | 26 | 42 |
| Seton + UST | Biron *et al.* 2019 | NR | 31 | NR | NR | NR | NR | NR | Cessation of drainage | Successful seton ablation | 88 | 33 |
| Seton + VED | Biron *et al.* 2019 | NR | 32 | NR | NR | NR | NR | NR | Cessation of drainage | Successful seton ablation | 61 | 15 |

^a^Median, range [unless stated otherwise].

^b^All setons removed before complete response was reached.

^c^Value was estimated based on a graph because it has not been mentioned in the text of the respective publication.

^d^Value for a median FU of 10 months (range 3–26 months).

^e^Value after 10 years.

^f^Value at end of the infliximab induction treatment (week 8–12 after first infusion).

^g^Trans-sphincteric/horseshoe fistula.

^h^Value at 18 months.

^i^IFX infusion ≤30 days post-seton.

^j^IFX infusion >30 days post-seton.

^k^Some setons were left in place owing to patient preference.

ADA, adalimumab; CER, certolizumab; Def., definition; EUS, endoscopic ultrasound; FU, follow-up; IFX, infliximab; NR, not reported; Pts, patients; Tx, treatment; VED, vedolizumab.

## Supplementary Table 5. Studies reporting rates of fistula recurrence

| **Intervention** | **Study** | **Ref.** | **Recurrent or ND fistula** | **Seton duration^a^ [months]** | **Recurrence definition [as stated in paper]** | **Pts [*n*]** | **Rate [%]** | **Time to recurrence^a^ [months]** |
| --- | --- | --- | --- | --- | --- | --- | --- | --- |
| Seton | Faucheron *et al.* 1996 | 36 | Mixed | 12 [mean], 4–60 | NR | 18 | 41 | 22, 3–55 [mean, range] |
| Seton | Felton *et al*. 2020 | 37 | NR | NR | Recurrence of symptoms | 22 | 27^d^ | NR |
| Seton | Felton *et al*. 2020 | 37 | NR | NR | Recurrence of symptoms | 31 | 68^e^ | NR |
| Seton | Higashi *et al*. 2009 | 44 | NR | NR | Recurrence after seton removal; patients had not reached complete response yet | 86 | 14 | NR |
| Seton | Motamedi *et al*. 2021 | 51 | Mixed | Initial seton: 10, 6–17 [IQR] | Reintervention after initial seton removal for recurrent infections | 37 | 68 | NR |
| Seton | Papaconstantinou *et al*. 2017 | 53 | Mixed | NR | Recurrence of perianal external fistula orifice with or without the presence of abscess during follow-up; seton removed prior to recurrence | 25 | 4 | NR |
| Seton | Park *et al*. 2021 | 55 | NR | 14, 1.4–41.4 | Recurrent pain with perianal swelling or discharge after complete healing | 17 | 6 | NR |
| Seton | Park *et al*. 2021 | 55 | NR | 14, 1.4–41.4 | Recurrent pain with perianal swelling or discharge after complete healing | 25 | 8 | NR |
| Seton | Reddy *et al.* 2016 | 56 | NR | 298.2 ± 277.3 days [mean ± SD]^b^; Pts with no recurrence: 280.3 ± 213.3 days; Pts with recurrence: 145 ± 59.8 days | NR | 57 | 26 | NR |
| Seton | Sciaudone *et al.* 2010 | 64 | NR | NR | Reopening of external fistula tracks with active drainage or the development of a perianal abscess at the site of original fistula | 7 | 43 | 3.5 ± 0.5 [mean ± SD] |
| Seton | Shinozaki *et al*. 2002 | 67 | NR | Long-term | Presence of fistula discharge after closure of all the fistulas | 21 | 33 | NR |
| Seton | Takesue *et al*. 2002 | 73 | NR | 11, 6–18 | NR | 9 | 33 | 61, 19–122 |
| Seton | Williams *et al.* 1991 | 80 | NR | Long-term | Discharge or an abscess developing in the same region as the original fistula | 23 | 9 | 6 months to 3.5 years |
| Seton + anti-TNF [IFX/ADA] | Foo *et al*. 2021 | 38 | NR | NR | Recurrence of active fistulizing disease at 12-month review after seton removal | 13 | 0^g,h^ | NR |
| Seton + anti-TNF [IFX/ADA] | Foo *et al*. 2021 | 38 | NR | NR | Recurrence of active fistulizing disease at 12-month review after seton removal | 5 | 100^h,i^ | NR |
| Seton + anti-TNF [IFX/ADA] | Kotze *et al*. 2014 | 49 | NR | 6, 1–36 | Reactivation of previous healed tracks [recurrent drainage after complete perianal remission] | 41 | 10 | 74.8 [mean] |
| Seton + anti-TNF [IFX/ADA] | Lin *et al*. 2016 | 50 | NR | NR | Fistula reappearance after complete healing or perianal abscess reappearance after the completion of drainage | 65 | 17 | 5.5 ± 6.5 [mean ± SD] |
| Seton + anti-TNF [IFX/ADA] | Park *et al.* 2021 | 54 | Mixed | NR | Cumulative fistula recurrence [relapse of discharge and perianal symptoms, including pain and swelling after closure of fistulous tract] | 39 | 0 | NR |
| Seton + IFX | Gao *et al.* 2013 | 41 | NR | NR | Relapse [no response prior to relapse] | 14 | 29 | NR |
| Seton + IFX | Hotokezaka *et al.* 2011 | 45 | NR | 20.8 ± 4.6 [mean ± SD]^c^ | Recurrence after complete response [no seton removal before response] | 15 | 7 | 19 [median] |
| Seton + IFX | Hukkinen *et al.* 2014 | 46 | Mixed | 8, 5–10.5 [IQR] | Appearance of active drainage from the fistula track after initial response or development of a new fistula | 13 | 23 | 14, 8–34 |
| Seton + IFX | Jeon *et al*. 2019 | 47 | NR | NR | Development of new fistulas or appearance of discharge from a former tract | 49 | 6^e^ | 9.5, 5–22^b^ |
| Seton + IFX | Jeon *et al*. 2019 | 47 | NR | NR | Development of new fistulas or appearance of discharge from a former tract | 27 | 11^f^ | 9.5, 5–22^b^ |
| Seton + IFX | Regueiro *et al*. 2003 | 57 | ND | Up to 2–4 weeks after 2nd IFX infusion | Reopening of the external fistula track with active drainage | 6 | 50 | 13 [mean] |
| Seton + IFX | Regueiro *et al*. 2003 | 57 | ND | Up to 2–4 weeks after 2nd IFX infusion | Reopening of the external fistula track with active drainage noted on history and examination | 9 | 44 | 13.5 [mean] |
| Seton + IFX | Sciaudone *et al*. 2010 | 64 | NR | NR | Reopening of external fistula tracks with active drainage or the development of a perianal abscess at the site of the original fistula | 11 | 18 | 10.1 ± 2.4 [mean ± SD] |
| Seton + IFX | Talbot *et al*. 2005 | 74 | Mixed | NR | NR | 10 | 0 | NR |
| Seton + IFX | Tanaka *et al*. 2010 | 75 | NR | Until 3rd IFX infusion: 54%; 4th: 9%; 5th: 18%; 8th: 18% | Relapse after complete response | 10 | 10 | NR |
| Seton + IFX | Tougeron *et al.* 2009 | 76 | NR | NR | Discharge from a previously closed or non-productive fistula | 13 | 15 | 4–7 [range] |

^a^Median, range [unless stated otherwise]. ^b^Value available only for the overall patient group. ^c^After the first IFX infusion. ^d^No imaging [MRI or TRUS] before seton removal. ^e^IFX infusion ≤30 days post-seton. ^f^IFX infusion >30 days post-seton. ^g^Patients with PCDAI <10. ^h^Describes a specific case with a low patient number with highly variable rates between groups. ^i^Patients with PCDAI >10.

ADA, adalimumab; IFX, infliximab; IQR, interquartile range; MRI, magnetic resonance imaging; ND, newly diagnosed; NR, not reported; PCDAI, Pediatric Crohn's Disease Activity Index; Pts, patients; SD, standard deviation; TNF, tumor necrosis factor; TRUS, transrectal ultrasound.

## Supplementary Table 6. Studies reporting fistula-related reintervention rates and other outcomes

| **Intervention** | **Study** | **Fistula type** | **Ref.** | **Pts [*n*]** | **New fistula/abscess formation rate [%]** | **Recurrent abscess/ local sepsis rate [%]** | **Reintervention [definition as stated in paper] [%]** |
| --- | --- | --- | --- | --- | --- | --- | --- |
| Seton | Banayan *et al*. 2016 | NR | 30 | 41 | NR | NR | Additional seton placement: 71 |
| Seton | Galis-Rozen *et al*. 2010 | Complex | 40 | 17 | New fistula: 12  New abscess: 47 | NR | Seton relocation due to displacement: 29  Second seton inserted due to additional external opening: 12 |
| Seton | Gklavas *et al.* 2021 | NR | 42 | 66 | NR | NR | Reoperation during follow-up: 29 |
| Seton | Higashi *et al*. 2009 | Mixed | 44 | 86 | NR | NR | Stoma: 5 |
| Seton | Koganei *et al.* 1995 | Complex | 48 | 13 | NR | NR | Redrainage: 46  Lay-open surgery: 8 |
| Seton | Motamedi *et al.* 2021 | Mixed | 51 | 43 | NR | NR | Reintervention for recurrent infections after initial seton removal: 68 |
| Seton | Mujukian *et al*. 2020 | Mixed | 52 | 23 | New fistula: 26  New abscess: 26 | NR | Postoperative ileostomy: 26  Fecal diversion: 26  New seton: 9 |
| Seton | Reddy *et al.* 2016 | NR | 56 | 57 | New fistula: 9 | NR | Additional seton placement: 46  Diversion due to failed seton treatment: 14 |
| Seton | Schaad *et al.* 2021 | NR | 60 | 98 | NR | NR | One or more additional surgeries: 50 |
| Seton | Sciaudone *et al*. 2010 | Complex | 64 | 10 | NR | NR | Proctectomy or diversion due to fistula recurrence or worsened clinical symptoms: 20 |
| Seton | Scott *et al.* 1996 | Mixed | 65 | 27 | NR | NR | Proctectomy: 11 |
| Seton | Sebastian *et al.* 2018 | Mixed | 22 | 96 | NR | NR | Seton reinsertion after seton removal: 34 |
| Seton | Shinozaki *et al.* 2002 | Mixed | 67 | 39 | NR | NR | Fecal diversion: 23  Stoma: 23 |
| Seton | Stellingwerf *et al.* 2020 | NR | 71 | 24 | New abscess: 25 | NR | Reintervention due to abscess: 8 |
| Seton | Stellingwerf *et al.* 2020 | NR | 71 | 17 | New abscess: 18 | NR | Reintervention due to painful fissure or loosening of connection: 18 |
| Seton | Sugita *et al.* 1995 | Mixed | 72 | 21 | NR | Recurrent abscess: 43 | Redrainage for recurrent fistulous abscess: 43  Lay-open after good result: 5  Fistulectomy after good result: 5 |
| Seton | Takesue *et al.* 2002 | Mixed | 73 | 32 | NR | Recurrent abscess: 7  Sepsis recurrence: 31 | Complementary drainage by insertion of an additional seton due to sepsis reappearance: 31  Malecot catheter insertion due to recurrence: 25  Hartmann’s operation due to longstanding severe proctitis and incontinence: 3  Proctocolectomy due to cancer of the fistula: 3 |
| Seton | Wasmann *et al.* 2020 | Complex | 78 | 15 | NR | NR | Reintervention: 67 |
| Seton | Wasmann *et al*. 2020 | Complex | 78 | 20 | NR | NR | Reintervention: 40 |
| Seton | White *et al.* 1990 | Complex | 79 | 10 | New abscess: 10 | Recurrent abscess: 10 | Incision and drainage of abscesses: 20  Repeated dilations of a rectal stricture: 10 |
| Seton | Williams *et al.* 1991 | Complex | 80 | 23 | NR | NR | Proctocolectomy for severe colonic disease required after fistula tract remained quiescent without abscess but did not close: 9  Proctectomy due to seton failure and because of progressive perianal and rectal CD: 13 |
| Seton + ADA | Solina *et al.* 2016 | Complex | 69 | 58 | NR | NR | Stoma: 7 |
| Seton + anti-TNF (IFX/ADA) | Lin *et al.* 2016 | Mixed | 50 | 65 | NR | NR | NR |
| Seton + Anti-TNF | Wasmann *et al*. 2020 | Complex | 78 | 15 | NR | NR | Reintervention: 40 |
| Seton + Anti-TNF | Wasmann *et al*. 2020 | Complex | 78 | 21 | NR | NR | Reintervention: 43 |
| Seton + anti-TNF (IFX/ADA) | Yardimci *et al.* 2016 | Mixed | 81 | 27 | New abscess: 11 | NR | NR |
| Seton + IFX | Bouguen *et al*. 2013 | Mixed | 33 | 97 | NR | NR | New seton drainage: 27 |
| Seton + IFX | Hotokezaka *et al.* 2011 | Mixed | 45 | 20 | NR | NR | NR |
| Seton + IFX | Regueiro *et al.* 2003 | Mixed | 57 | 9 | NR | NR | NR |
| Seton + IFX | Sciaudone *et al*. 2010 | Complex | 64 | 14 | NR | NR | Proctectomy or diversion: 0 |
| Seton + IFX | Spradlin *et al.* 2008 | Complex | 70 | 5 | NR | NR | Repeat seton placement: 60  Repeat incision and drainage: 20  Fibrin glue injection: 40 |
| Seton + IFX | Tanaka *et al*. 2010 | NR | 75 | 14 | NR | NR | NR |
| Seton + IFX | Tougeron *et al*. 2009 | Mixed | 76 | 26 | New abscess: 12 | NR | Subsequent abscess and fistula drainage due to new abscess: 12  Defunctioning stoma: 4 |
| Seton + IFX/ADA/UST | Saigusa *et al.* 2018 | Mixed | 59 | 18 | NR | Sepsis recurrence: 61 | Additional seton drainage due to recurrent local sepsis: 61 |

ADA, adalimumab; CD, Crohn’s disease; IFX, infliximab; NR, not reported; Pts, patients; TNF, tumor necrosis factor; UST, ustekinumab.
